# Association Between Septal Implantation Level and Pacing Threshold Stability in Leadless Pacemaker Implantation

**DOI:** 10.3390/jcm15020468

**Published:** 2026-01-07

**Authors:** Dong-Hyeok Kim, Yeji Kim, Seung Woo Lee, Jeongmin Kang, Junbeom Park

**Affiliations:** 1 Division of Cardiology, Ewha Womans University Seoul Hospital, Seoul 07804, Republic of Korea; thomas76@ewha.ac.kr; 2 Division of Cardiology, Ewha Womans University Mokdong Hospital, Seoul 07985, Republic of Korea; win547@hotmail.com (S.W.L.); angelak6506@gmail.com (J.K.); newriser@naver.com (J.P.)

**Keywords:** pacemaker, leadless, threshold

## Abstract

**Background/Objective:** Leadless pacemakers (LPs, Micra™, Medtronic) offer a safe alternative to traditional transvenous systems. However, optimal implantation site within the right ventricular septum (RVS) and its effect on long-term pacing threshold stability remains under debate. The aim was to evaluate the relationship between pacing site within the RVS and pacing threshold stability following leadless pacemaker implantation. **Methods:** We retrospectively analyzed 36 patients who underwent LP implantation at two centers between 2022 and 2023. Patients were classified into two groups based on final device position by fluoroscopy: Group A (mid or upper RVS, *n* = 8) and Group B (low or apical RVS, *n* = 28). Pacing threshold, QRS duration, and left ventricular ejection fraction (LVEF) were assessed over 6 months. **Results:** At the 6-month follow-up, Group A demonstrated significantly lower and more stable pacing thresholds compared to Group B (0.57 ± 0.09 mV vs. 1.55 ± 0.97 mV, *p* < 0.001). No significant differences were observed in QRS duration or LVEF changes between groups. Echocardiography did not reveal new-onset tricuspid regurgitation. **Conclusions:** Given the small sample size, particularly in the mid/high septal group, these findings should be interpreted as hypothesis-generating and require confirmation in larger prospective studies. These findings highlight the importance of careful anatomical targeting during LP implantation. Prospective studies incorporating advanced imaging are warranted to confirm these results and evaluate long-term clinical outcomes.

## 1. Introduction

Leadless pacemakers (LPs) have rapidly evolved over the past decade and are now widely recognized as an important alternative to conventional transvenous pacing systems. The Micra™ Transcatheter Pacing System (Medtronic), the first widely adopted leadless pacemaker, has demonstrated excellent safety and durability, with markedly lower rates of infection, lead dislodgement, and pocket-related complications compared with transvenous systems [1,2]. As implantation techniques have improved and long-term registry data have accumulated, the clinical use of LPs has broadened to include elderly patients, individuals with limited venous access, and those at high risk for device-related infections [3].

Despite these advancements, the long-term performance of leadless pacemakers depends heavily on the stability of electrical parameters—particularly pacing thresholds, which directly influence battery longevity and the need for early device replacement. While the right ventricular septum (RVS) is generally preferred over the apex due to reduced risk of perforation and more favorable myocardial characteristics, the optimal septal region for implantation remains uncertain. Several recent multicenter analyses and advanced imaging studies have reported that mid- or high-septal implantation may be associated with improved electrical performance, more stable thresholds, and potentially more physiological ventricular activation compared with lower septal or apical positions [4,5,6,7].

Growing evidence suggests that these differences may stem from the heterogeneous microstructure of the RV septum. Upper and mid-septal areas typically have thicker myocardium, more favorable trabeculation patterns for device fixation, and closer proximity to physiologic conduction pathways, all of which may contribute to stable long-term capture thresholds [8,9]. In contrast, pacing in the lower RVS or near the apex may be more affected by myocardial motion, anatomical curvature, and micro-dislodgement forces that can compromise chronic electrical performance.

Furthermore, recent guideline updates—including the 2023 ESC Guidelines on Cardiac Pacing and the 2023 HRS Expert Consensus—have increasingly emphasized anatomical precision and septal targeting when implanting leadless pacemakers, although they do not yet define the optimal sub-region within the septum [10,11]. As LP implantation becomes more frequent in routine practice, research focusing on the impact of anatomical site selection on chronic device behavior has become increasingly important.

However, despite growing interest, clinical studies directly comparing pacemaker thresholds according to detailed septal level (high/mid vs. low/apical) remain scarce. Our study explores whether different septal sub-regions may be associated with differences in pacing threshold stability. Therefore, the present study aims to clarify the association between the final pacing location within the RVS and long-term pacing threshold stability using real-world data from two tertiary centers. These findings may provide important insights that help refine procedural strategies and improve long-term outcomes for patients receiving leadless pacemakers.

## 2. Methods

### 2.1. Study Design and Population

This was a retrospective, dual-center observational study conducted at Ewha Womans University Medical Center and one affiliated hospital between January 2022 and December 2023. A total of 36 patients who underwent successful leadless pacemaker (LPs, Micra™ Transcatheter Pacing System, Medtronic) implantation were enrolled. Patients were divided into two groups based on the final pacing location confirmed by fluoroscopic guidance at the time of implantation:

Group A: Mid or high right ventricular septum (RVS) pacing (*n* = 8).

Group B: Low or Apical RVS pacing (*n* = 28).

**Figure 1:**
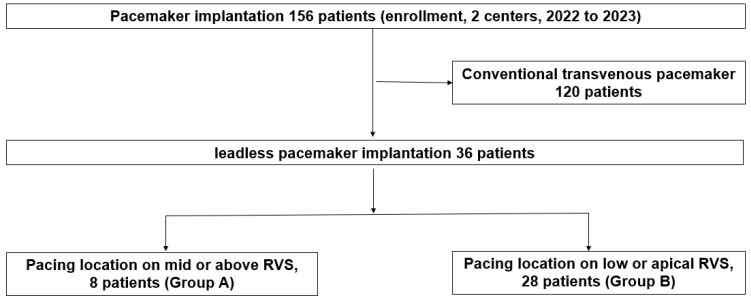
Study flow diagram. Flow diagram summarizing patient enrollment and allocation. A total of 156 patients underwent pacemaker implantation across two centers between 2022 and 2023. Among these, 36 received a leadless pacemaker, while 120 received a conventional transvenous pacemaker. Leadless pacemaker recipients were categorized by pacing site into Group A (mid or upper right ventricular septum) and Group B (low or apical septum). RVS: right ventricular septum.

Figure 1 shows that during follow-up (mean 6 months), we analyzed pacing threshold and QRS duration on electrocardiography (ECG) after implantation.

### 2.2. Procedure for Implantation of AF Ablation

**Figure 2:**
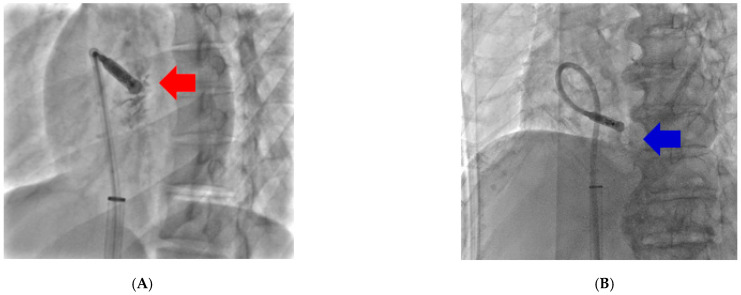
Fluoroscopic Views of Leadless Pacemaker Implantation Sites. (**A**) Fluoroscopic image demonstrating leadless pacemaker fixation at the mid-to-upper right ventricular septum (Group A). (**B**) Leadless pacemaker anchored at the low or apical septum (Group B). Arrows indicate the implantation site in each corresponding panel.

All procedures were performed under local anesthesia and fluoroscopic guidance via a femoral venous approach. The Micra delivery catheter was advanced into the right ventricle, and positioning was attempted preferentially on the septal wall. The location was determined based on right anterior oblique (RAO) and left anterior oblique (LAO) projections. Adequate electrical parameters (pacing threshold ≤2.0 V at 0.24 ms, sensing >5 mV, and impedance 300–1500 ohms) were confirmed before final deployment. Device repositioning was performed at the operator’s discretion when thresholds exceeded 2.0 V or if fixation was inadequate. Figure 2 shows the approach location of leadless pacemaker. Patients with pacing location on mid or high RVS was 8 (Group A). Other patients with pacing location on low or apical RVS was 28 (Group B). The Micra™ Transcatheter Pacing System was manufactured by Medtronic (Minneapolis, MN, USA).

### 2.3. Postprocedural Follow-Up

Follow-up evaluations were conducted at 1, 3, and 6 months post-implantation. At each visit, surface electrocardiograms (ECGs) were recorded and pacing thresholds, impedance, and sensing values were measured using device interrogation. Transthoracic echocardiography (TTE) was performed pre- and post-implantation to assess changes in left ventricular ejection fraction (LVEF). The primary endpoint was pacing threshold stability, defined as the absolute change in threshold from implantation to final follow-up. Secondary endpoints included changes in LVEF and QRS duration.

### 2.4. Statistical Analysis

Continuous variables were expressed as mean ± standard deviation (SD) and compared using the independent samples t-test for normally distributed variables or the Mann–Whitney U test for non-normally distributed data. Categorical variables were expressed as counts (percentages) and analyzed using the chi-square or Fisher’s exact test, as appropriate. A *p*-value < 0.05 was considered statistically significant. All statistical analyses were performed using IBM SPSS Statistics software, version 26.0 (IBM Corp., Armonk, NY, USA). Given the unequal group sizes, we performed adjusted and sensitivity analyses to assess the robustness of threshold comparisons. Because of the limited sample size and the unequal distribution between groups, particularly the small number of patients in the mid/high septal group, the statistical analyses were not intended to establish definitive correlations. Therefore, the results should be interpreted as exploratory, and no causal inference was assumed.

### 2.5. IRB and Ethical Approval

This study was reviewed and approved by the Institutional Review Board (IRB) of Ewha Womans University Seoul Hospital (SEUMC IRB No. 2025-06-073-002). According to the official approval notification dated 18 July 2025, the board granted full approval through an expedited review process, authorizing the conduct of this investigation for the designated research period from 18 July 2025 to 17 July 2026. The IRB confirmed that the submitted revisions and clarifications adequately addressed all review comments, and therefore the study was approved for a one-year duration, with mandatory continuing review before the expiry date. As stated in the approval letter, the study complies with the Bioethics and Safety Act of Korea, the Medical Device Act, the Declaration of Helsinki, and ICH-GCP guidelines, and research activities may begin only after formal IRB approval. All procedures involving human participants were performed in accordance with institutional and national ethical standards.

## 3. Results

### 3.1. Baseline Characteristics

A total of 36 patients underwent leadless pacemaker (LPs) implantation at two tertiary centers between 2022 and 2024. Based on the fluoroscopically guided pacing site, patients were categorized into two groups: Group A (mid or high right ventricular septum [RVS], *n* = 8) and Group B (low or apical RVS, *n* = 28). Table 1 demonstrates that there were no statistically significant differences between the two groups in terms of age (58.8 ± 17.3 vs. 70.9 ± 13.4 years, *p* = 0.100), sex distribution (male: 62.5% vs. 50.0%, *p* = 0.695), or initial QRS duration (118.2 ± 35.1 ms vs. 119.9 ± 37.7 ms, *p* = 0.909). The initial implantation threshold was lower in Group A compared to Group B, though not statistically significant (0.43 ± 0.20 mV vs. 0.58 ± 0.51 mV, *p* = 0.238).

### 3.2. Follow-Up Pacing Thresholds

**Figure 3:**
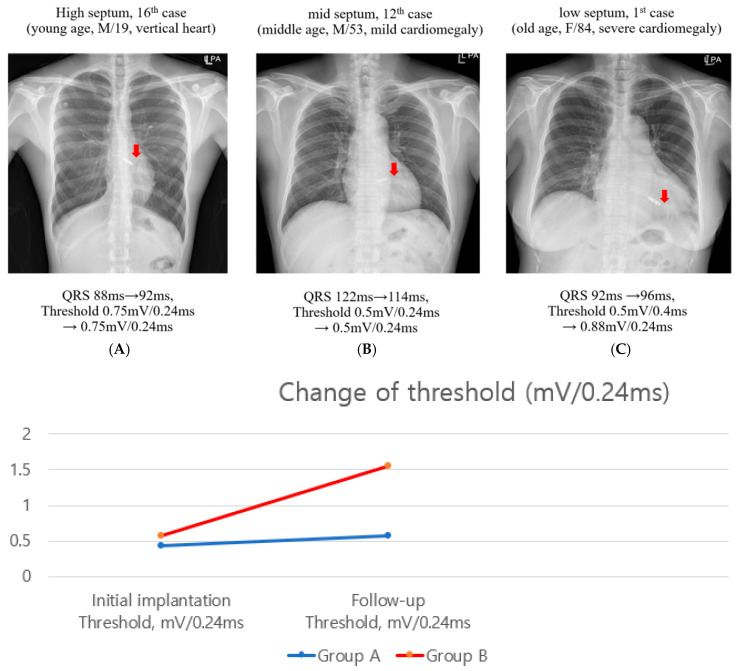
Representative Chest Radiographs and Threshold Changes According to Pacing Site. (**A**) High-septal pacing example from a young patient with a vertically oriented heart. (**B**) Mid-septal pacing example in a middle-aged patient with mild cardiomegaly. (**C**) Low-septal pacing example in an elderly patient with significant cardiomegaly. (**Below**): longitudinal change in pacing threshold (mV/0.24 ms) comparing Group A and Group B from implantation to follow-up. Red arrow shows the position of leadless pacemaker.

At a mean follow-up of 6 months, a significant difference in pacing threshold was observed between the groups (Table 2). Group A demonstrated lower pacing thresholds compared with Group B (mean difference −0.98 mV; 95% CI −1.45 to −0.51; *p* < 0.001). Figure 3 illustrates representative cases of pacing threshold stability according to pacing site. The high septal pacing case maintained a consistent threshold of 0.75 mV, and mid-septal pacing demonstrated stable 0.5 mV over time, whereas low septal pacing cases showed variable or elevated thresholds over follow-up. Although a difference in pacing thresholds was observed between groups, the limited number of patients, especially in the mid/high septal group, precludes robust correlation analyses and definitive statistical conclusions. During the follow-up period, no patients required device re-intervention or early system replacement. Formal patient-reported outcome measures and long-term device longevity data were not systematically collected in this retrospective cohort.

### 3.3. Changes in Echocardiographic and Electrical Parameters

There was no significant change in left ventricular ejection fraction (LVEF) and electrocardiogram (ECG) after implantation in either group (Table 2). Group A showed a slight increase in LVEF (from 61.05 ± 8.39% to 63.68 ± 8.68%), while Group B showed a small decrease (from 64.42 ± 6.32% to 64.08 ± 5.50%, *p* = 0.600). The change in LVEF between groups was not statistically significant (2.33 ± 2.34% vs. −0.32 ± 1.22 ms, *p* = 0.129). Tricuspid regurgitation (TR) was not found in any case. Furthermore, the change of ECG QRS duration between groups was not statistically significant (6.0 ± 12.70 vs. 13.5 ± 42.8 ms, *p* = 0.791).

## 4. Discussion

### 4.1. Main Findings

This retrospective analysis should be interpreted as a hypothesis-generating study. Although differences in pacing threshold stability were observed according to septal implantation level, the small sample size—particularly in the mid/high septal group—limits statistical power and precludes definitive correlation or causal inference. Our results showed a clear and consistent pattern: devices implanted in the mid or high RVS maintained significantly lower and more stable pacing thresholds throughout mid-term follow-up, whereas implants positioned in the low or apical RVS were more likely to exhibit elevated or fluctuating thresholds over time.

This distinction persisted despite comparable baseline characteristics and similar acute implantation thresholds, suggesting that intrinsic regional myocardial properties, rather than procedural variation or patient factors, drive these differences. The findings highlight that the septal level—not simply avoiding the apex—plays a central role in achieving durable electrical stability. The mid/high septal regions may provide more favorable tissue characteristics for device fixation and chronic capture, including thicker myocardium, reduced mechanical motion, and more uniform engagement with pacing vectors.

Collectively, these observations reinforce a growing body of literature advocating for an anatomically targeted approach to leadless pacemaker implantation. They also emphasize the need for more refined procedural strategies, where operators intentionally aim for mid-to-upper septal zones to optimize chronic pacing outcomes and potentially prolong device longevity. Our study provides real-world evidence supporting this shift and offers practical insights that may guide implantation techniques in contemporary clinical practice.

### 4.2. Comparison of Recent Guidelines

Recent data from the Micra Global Registry and other multicenter studies have highlighted that high or mid-septal implantation can lead to improved electrical performance and reduced long-term energy consumption, potentially extending battery longevity [2,3]. Our results align with these findings, as the follow-up threshold in the low or apical RVS group was significantly higher than in the mid or upper RVS group (1.55 ± 0.97 mV vs. 0.57 ± 0.09 mV, *p* < 0.001). This reinforces the notion that leadless pacemaker implantation should be guided by both anatomical safety and long-term pacing efficiency.

Importantly, the 2023 ESC Guidelines on Cardiac Pacing and Cardiac Resynchronization Therapy and the 2023 HRS Expert Consensus Statement on Conduction System Pacing emphasize careful selection of the pacing site in leadless systems, particularly in light of their fixed positioning and lack of lead maneuverability [5,6]. These guidelines recommend septal rather than apical implantation for most patients, citing the potential for lower thresholds and more physiological activation. However, they do not yet clearly differentiate between mid or high- and low-septal positions—an area in which our study contributes the first meaningful data in Korean practical procedure [7,8].

### 4.3. Pacing Threshold Change of Mid-Term Follow-Up

Another consideration is the threshold change over time. Previous studies noted that elevated acute thresholds may decrease within the first few weeks’ post-implantation, especially if the acute threshold is ≤2.0 V [9]. While our data show some stability in thresholds regardless of initial values, the superior stability in mid-to-high septal implants suggests that anatomical myocardial properties—such as wall thickness, trabeculation, and proximity to conduction fibers—may influence chronic pacing performance.

### 4.4. Clinical Studies and What’s New Findings on This Study

Leadless pacemakers represent a transformative advancement in cardiac rhythm management, and optimal implantation site selection is an important consideration in the procedure [11,12]. In this small retrospective cohort, septal implantation level was associated with differences in pacing threshold stability, and these findings should be regarded as hypothesis-generating only.

Beyond traditional concerns of safety and electrical stability, the pacing site may influence myocardial remodeling, device–tissue interaction, and valve function [13,14]. Anatomical heterogeneity across the right ventricular septum—including wall thickness, trabecular complexity, and conduction fiber distribution—contributes to differing mechanical and electrophysiological dynamics among septal regions [15,16]. These distinctions contextualize why mid- or high-septal sites may promote more favorable pacing thresholds and long-term stability [17,18].

Recent multicenter studies and registry analyses reinforce the importance of septal targeting [19,20]. Cantillon et al. demonstrated that microstructural myocardial characteristics influence chronic pacing capture [21], while Mittal et al. emphasized that optimal septal engagement can reduce mechanical stress on device fixation components [22]. Although leadless systems do not provide conduction system pacing, upper septal implantation may approximate more physiological activation and minimize dyssynchrony [23,24]. While our cohort did not show significant QRS narrowing, accumulating evidence suggests that subtle activation benefits may manifest in larger or more detailed electrophysiologic assessments [25,26]. Previous studies in conventional transvenous pacemakers have not demonstrated consistent differences in chronic pacing thresholds between RVA and septal pacing sites. Therefore, no definitive physiological mechanism can be inferred from these data. In contrast, leadless pacemakers rely on direct myocardial fixation and device–tissue interaction, which formed the basis of our exploratory hypothesis rather than an assumption of physiological superiority.

Taken together, these data support a paradigm shift toward anatomically informed, imaging-guided site selection for leadless pacemaker implantation [18,27]. Integration of advanced imaging—3D transthoracic echocardiography, CT-based myocardial mapping, or intracardiac echocardiography—could improve precision in targeting favorable septal regions [28,29]. Future investigations should incorporate RV functional metrics such as RV strain, TAPSE, and FAC to clarify downstream hemodynamic consequences of pacing site differences [30,31].

Finally, the possibility of subtle tricuspid valve interaction warrants systematic longitudinal imaging [32,33]. Although our cohort showed no clinically significant TR, several observational studies have reported structural changes after LP implantation [14,34]. Multi-modality imaging and long-term follow-up will be essential to elucidate these risks and develop preventative strategies [12,35].

From a clinical perspective, these findings suggest that when feasible, operators may consider targeting mid- to high-septal regions during leadless pacemaker implantation to potentially enhance pacing threshold stability. While septal implantation is already standard practice for safety reasons, further refinement of septal targeting may contribute to improved long-term device performance. Recent data have further emphasized the importance of implantation site selection in leadless pacing systems. A contemporary analysis published in Heart Rhythm demonstrated that septal positioning was associated with favorable electrical performance and reduced complication rates, reinforcing the need for anatomically informed implantation strategies [36].

### 4.5. Study Limitations

A major limitation of this study is the small and imbalanced sample size, particularly the limited number of patients in the mid/high septal group. This constraint reduces statistical power and limits the ability to detect statistically significant correlations. Consequently, the findings should be regarded as exploratory and hypothesis-generating.

Although our sample size is limited, the findings underscore the need for fluoroscopic or intracardiac echocardiographic guidance to precisely target ideal septal regions during implantation. In addition, advanced tools such as three-dimensional electro-anatomical mapping or pre-procedural CT integration may aid in optimizing pacing sites in complex cases.

Retrospective, collected data from only 2 hospitals showed no clinical benefit was found with transthoracic echocardiogram (TTE) parameter including LVEF and QRS duration. And data of QRS duration change after pacing is not enough.

While our study provides meaningful insight into the relationship between pacing location and threshold stability in leadless pacemakers, several limitations must be acknowledged.

First, despite avoiding transvalvular leads, leadless pacemakers are not entirely free from the risk of structural complications such as tricuspid valve regurgitation (TR). Recent observational studies have reported new-onset or worsened TR after LP implantation, especially when the device is positioned near the sub-valvular apparatus or anchored in a way that impinges upon leaflet motion or chordal structures [10,11]. Although our cohort did not exhibit clinically significant TR during follow-up, systematic assessment using advanced imaging modalities such as three-dimensional (3D) transthoracic echocardiography or transesophageal echocardiography was not performed. Therefore, subtle or subclinical changes may have been underrecognized.

Second, this study did not include detailed right ventricular (RV) functional parameters, which are increasingly recognized as critical markers in evaluating long-term cardiac performance post-pacing. Echocardiographic measures such as RV fractional area change (FAC), tricuspid annular plane systolic excursion (TAPSE), RV free wall strain, and RV longitudinal strain provide valuable information on RV adaptation to pacing and should be incorporated in future investigations. Since the RV is particularly susceptible to mechanical and electrical remodeling due to altered activation patterns, especially in cases of apical pacing, these parameters would have enhanced the functional assessment of pacing site selection.

Third, our data were limited to conventional 2D TTE and standard device interrogation, and the retrospective nature of the study limits causal inference. Integration of multimodality imaging—including 3D echo, CT-based myocardial anatomy, and electro-anatomical mapping—would allow for more precise characterization of both device-tissue interaction and functional consequences.

Finally, clinical outcomes such as device longevity, patient-reported symptomatic improvement, and long-term re-intervention rates were not systematically assessed. These endpoints should be incorporated into future prospective studies to better define the clinical relevance of septal implantation level.

## 5. Conclusions

This data showed that pacing threshold of leadless pacemakers (LPs) after implantation is related to pacing location and mid or high right ventricular septum (RVS) is better than low RVS. These findings should be regarded as hypothesis-generating only. Larger, prospective studies with sufficient statistical power are required to validate this potential association and clarify its clinical relevance.

## Figures and Tables

**Table 1:** Baseline characteristics.

	Group A, *n* = 8 (Mid or Above RVS)	Group B, *n* = 28 (Lower RVS)	*p* Value
Age (years)	58.8 ± 17.3	70.9 ± 13.4	0.100
Sex, male, *n* (%)	5 (62.5%)	14 (50.0%)	0.695
Pacemaker indication AV block, *n* (%)	4 (30.7%)	5 (19.2%)	0.414
Initial QRS duration, ms	118.2 ± 35.1	119.9 ± 37.7	0.909
Initial implantation Threshold, mV/0.24 ms	0.43 ± 0.20	0.58 ± 0.51	0.238
Follow-up Threshold, mV/0.24 ms	0.57 ± 0.09	1.55 ± 0.97	<0.001

Values are expressed as means SDs and numbers (percentages). RVS: right ventricular septum, AV: atrioventricular.

**Table 2:** Comparison of mid or above RVS and lower RVS pacing.

	Group A (Mid or Above RVS)	Group B (Lower RVS)	*p* Value
LVFE (%)			
Pre-implantation	61.05 ± 8.393	64.42 ± 6.32	0.584
Post-implantation	63.68 ± 8.68	64.08 ± 5.50	0.600
Change of LVEF	2.33 ± 2.34	−0.32 ± 1.22	0.129
QRS duration (ms)			
Pre-implantation	118.2 ± 35.1	119.9 ± 37.7	0.909
Post-implantation	111.6 ± 22.4	123.5 ± 41.8	0.279
Change of QRS duration	6.0 ± 12.7	13.5 ± 42.8	0.791

Values are expressed as means SDs and numbers (percentages). RVS: right ventricular septum, AV: atrioventricular.

## Data Availability

The data presented in this study are available from the corresponding author upon reasonable request.
